# A Temporal Association between Accumulated Petrol (Gasoline) Lead Emissions and Motor Neuron Disease in Australia

**DOI:** 10.3390/ijerph121215047

**Published:** 2015-12-19

**Authors:** Mark A. S. Laidlaw, Dominic B. Rowe, Andrew S. Ball, Howard W. Mielke

**Affiliations:** 1 Centre for Environmental Sustainability and Remediation (EnSuRe), School of Applied Sciences, RMIT University, PO Box 71, Bundoora, Victoria 3083, Australia; andy.ball@rmit.edu.au; 2 Department of Neurology, Faculty of Medicine and Health Sciences, Suite 204, 2 Technology Place Macquarie University, Sydney 2109, Australia; dominic.rowe@mq.edu.au; 3 Department of Pharmacology, Tulane University School of Medicine, 1430 Tulane Avenue, New Orleans, Louisiana, LA 70112, USA; hmielke@tulane.edu

**Keywords:** amyotrophic lateral sclerosis, bone lead, motor neuron disease, poisoning, petrol, gasoline, soil

## Abstract

Background: The age standardised death rate from motor neuron disease (MND) has increased from 1.29 to 2.74 per 100,000, an increase of 112.4% between 1959 and 2013. It is clear that genetics could not have played a causal role in the increased rate of MND deaths over such a short time span. We postulate that environmental factors are responsible for this rate increase. We focus on lead additives in Australian petrol as a possible contributing environmental factor. Methods: The associations between historical petrol lead emissions and MND death trends in Australia between 1962 and 2013 were examined using linear regressions. Results: Regression results indicate best fit correlations between a 20 year lag of petrol lead emissions and age-standardised female death rate (*R*^2^ = 0.86, *p* = 4.88 × 10^−23^), male age standardised death rate (*R*^2^ = 0.86, *p* = 9.4 × 10^−23^) and percent all cause death attributed to MND (*R*^2^ = 0.98, *p* = 2.6 × 10^−44^). Conclusion: Legacy petrol lead emissions are associated with increased MND death trends in Australia. Further examination of the 20 year lag between exposure to petrol lead and the onset of MND is warranted.

## 1. Introduction

Motor Neuron Disease (MND) is a general term for neurological disorders that affect motor neurons which are cells that control voluntary muscles including those affecting breathing speaking, swallowing and walking. Amyotrophic lateral sclerosis (ALS) is the commonest form of MND, affecting both corticomotor neurons in the cerebrum and the anterior horn cells in the brainstem and spinal cord [1]. This neurodegenerative disease results in increasing disability and, eventually, death.

### 1.1. Trends of Increases in Rates of Motor Neuron Disease (MND)

A review of the literature indicates that MND mortality rates have generally shown increasing trends. Veiga-Cabo *et al.* (1997) [2] documented that MND mortality rates increased in numerous countries between about 1970 and 1990. In France, Durrleman and Alperovitch (1989) [3] found that between the time periods of 1968–1971 and 1979–1982 the adjusted mortality rates (per 100,000) increased from 1.11 to 1.92 in men and from 0.63 to 1.12 in women. Similarly, Neilson *et al.* (1994) [4] found that in France they observed a consistent trend of rising ALS mortality between the years 1968 and 1990, a period during which crude mortality rose from 400 deaths in 1968 to 950 deaths in 1990. In Italy, Vittorio *et al.* (2003) [5] found that the incidence of MND increased from 1.07 to 2.19 per 100,000 population between 1964 and 1998. Seljeseth *et al.* (2000) [6] studied Norway and noted that between 1961 and 1994 the annual mortality of ALS increased from a rate of 1.38 to 2.54 per 100,000. More recently in Denmark, Seals *et al.* (2013) [7] indicated that age-adjusted mortality rates increased by an average of 3.0% annually between 1970 and 2009 and by an average of 2.1% annually after 1982. Okamoto *et al.* (2005) [8], observed in Japan that between 1995 and 2001 there were small increases in the number of ALS deaths (from 1249 to 1400 per year) and the crude mortality rates (from 1.00 to 1.10 per 100,000 population). In the United States, Noonan *et al.* (2005) [9] found that between 1969 and 1989 MND mortality rates increased 46% from 1.25 per 100,000 to 1.82 per 100,000. Noonan *et al.* (2005) [9] suggested that variations in motor neuron disease (MND) mortality by time, race/ethnicity, sex, and geography were consistent with the hypothesis that environmental exposures, combined with factors of genetic susceptibility, play a role in the development of MND.

While 10% of all MND cases are caused by monogenic mutations that are increasingly identified [10], the cause(s) of sporadic MND remains largely unknown. Most MND cases are believed to be caused by a combination polygenic variants and polymorphisms, environmental risk factors, and perhaps stochastic factors that exert their influence only in genetically susceptible individuals (Wang *et al*., 2014) [11]. Although many polymorphic gene variants and environmental factors (such as pesticides, heavy metals, trauma, smoking, and electrical injury) are associated with MND, none as yet are conclusively determined to cause MND [11]. Exposure to solvents and water bodies containing blue green algae are also considered risk factors for MND [12].

### 1.2. Lead and MND

Lead is a potent neurotoxin and there is a growing body of evidence that past lead exposure plays a possible causal role in a subset of MND patients [11]. Multiple studies have found positive associations between MND and lead in biological samples such as blood [13,14], urine [14], scalp hair [14,15] and spinal tissues [16]. Following exposure to lead, 90% to 95% of the lead is stored in bones [17,18,19] Lead accumulates in bone over time and bone lead is a good bio-indicator of historical lead exposure whereas measures of lead in blood reflect exposures to lead contemporaneously within about a month. In addition, qualitative questionnaire studies have identified that MND patients are more likely to have worked with metals [20] or had a history of exposure to lead [21,22]. 

Wang *et al.* (2014) [11] performed a meta-analysis regarding occupational lead and heavy metal exposure and the risk of developing MND. The results of the meta-analysis were based on nine articles and revealed that the odds of developing ALS were significantly higher among subjects with a history of occupational exposure to lead than among unexposed subjects (odds ratio—OR = 1.81; 95% confidence interval—CI = 1.39–2.36). A similar increase in the risk of ALS was also observed with heavy metals as the risk factor (OR = 2.13; 95% CI = 1.33–2.42). When Wang *et al.* (2014) [11] combined the nine articles focusing on lead exposure with the four articles focusing on exposure to all heavy metals an OR of 1.87 (95% CI = 1.57–2.33) was reported. The authors estimated five percent of sporadic MND may be attributable to lead.

There are two retrospective case-control studies that found an association between bone lead and MND. Kamel *et al.* (2002) [23] assessed the blood and bone lead in 109 MND cases and 256 controls. They found that the risk of MND was associated with self-reported occupational exposure to lead, OR = 1.9; 95% CI = 1.1–3.3), with a dose response for lifetime days of lead exposure. The risk of MND was associated with elevations of lead levels in both blood and bone. ORs were 1.9, CI = 1.4–2.6, for each µg/dL increase in blood lead, 3.6 (95% CI = 0.6–20.6) for each unit increase in log-transformed patella lead, and 2.3 (CI = 0.4–14.5) for each unit increase in log-transformed tibia lead. Kamel *et al.* (2003) [24] used the dataset from Kamel *et al.* (2002) [23] to investigate associations of MND with polymorphisms in the aminolevulinate dehydratase (*ALAD)* gene and the vitamin D receptor gene (VDR) and the influence of genotype on the previously observed association of MND with lead exposure. They concluded that genetic susceptibility conferred by polymorphisms in the *ALAD* gene may affect MND risk, possibly through a mechanism related to internal lead exposure (such as from bone lead). 

The Kamel *et al.* (2005) [25] study used both lead biomarkers from Kamel *et al.* (2002) [23] along with interview data to assess lead exposure to evaluate the role of genetic susceptibility to lead. Kamel *et al.* (2005) [25] observed that MND was associated with self-reported occupational lead exposure, with a dose response for cumulative days of exposure. MND was also associated with blood and bone lead levels, with a 1.9-fold increase in risk for each µg/dL increment in blood lead and a 2.3 to 3.6-fold increase for each doubling of bone lead. Furthermore, Kamel *et al.* (2005) [25] observed that a polymorphism in the *ALAD* gene was associated with a 1.9-fold increase in MND risk.

Eum *et al.* (2015) [26] measured bone lead, a biomarker of cumulative lead exposure, using K-shell-X-ray fluorescence in 100 neurologist-confirmed MND cases and 194 controls. They further examined the interaction with haemachromatosis genotype and found that the OR per 15.6 μg/g patella lead (interquartile range—IQR) was 8.24 (95% CI = 0.94–72.19) times larger among C282Y variant carriers, and 0.34 (95% CI = 0.15–0.78) times smaller among H63D variant carriers; results however were weaker for tibia lead.

### 1.3. Lead Exposure in Australia

In Australia, the majority of non-occupational lead exposure occurs in mining towns [27,28] and older urban areas [29]. Non-occupational lead exposure in mining towns, where relatively few Australians live, occurs primarily from lead contaminated soil dust [27,28]. Non-occupational exposure in urban areas, where most Australians live, results from lead in soil contaminated by leaded gasoline emissions and from house dust contaminated by soil and flaking, deteriorating exterior and interior paints [30]. 

Recent progress has been made in the understanding numerous chronic health effects associated with lead exposure. For example, there are strong associations between lead and a multitude of diseases such as autism [31,32] preeclampsia [33], developmental delays in children [34], heart disease [35], ADHD [36], dementia [37], and mental illness [38]. Nevertheless just when all of the health effects are thought to be fully understood there are new findings which point to yet another outcome previously unrecognized and missed [39] There are no known studies which have assessed the association between historical petrol lead emissions and increasing trends of MND deaths. 

**Figure 1:**
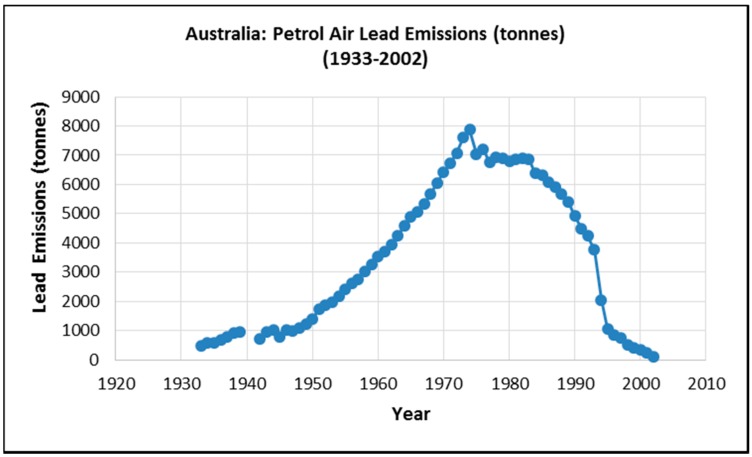
Calculated lead emissions from leaded petrol combustion in Australia [40].

### 1.4. Accumulated Lead

A major source of non-occupational lead exposure is from atmospheric emissions from leaded petrol. Historically this exposure source has been the principal driver of blood lead levels in the Australian population [40]. Leaded petrol accounted for a total 240,510 tonnes between the 1930s and 2002 [40]. As shown in Figure 1 lead in the atmosphere increased exponentially between about 1945 and 1975 when it peaked and remained elevated at high levels until about 1990 and then declined rapidly between about 1990 and 2002. Throughout this entire period the lead concentrations in the urban soils increased by 2002 to a maximum level. 

The accumulated lead emitted from automotive tailpipes was deposited into urban soils and dusts with a legacy of lead still present within the top 5 cm of soil. Because lead stays as a residue until geologic processes gradually cover or remove its expected “half-life” is approximately 700 years [41]. The primary aim of this study is to consider the association between non-occupational historical petrol lead emissions and age standardised death rates and percent of all cause MND deaths in Australia. 

## 2. Methods

Information about the MND deaths was collected on death certificates and certified by either a medical practitioner or a coroner. Registration of deaths is compulsory in Australia and is the responsibility of each state and territory Registrar of Births, Deaths and Marriages under jurisdiction specific legislation. On behalf of the Registrars, deaths data are assembled, coded and published by statistical agencies. These agencies have varied since 1900 and have included state based statistical offices, the Commonwealth Statistician's Office and the Commonwealth Bureau of Census and Statistics, now known as the Australian Bureau of Statistics (ABS). Cause of Death Unit Record File data are provided to the Australian Institute of Health and Welfare (AIHW) by the Registries of Births, Deaths and Marriages and the National Coronial Information System (managed by the Victorian Department of Justice) and include cause of death coded by the ABS (ICD10 code G12.2). The data are maintained by the AIHW in the National Mortality Database.

The Australian MND death rate is available for a fifty four year period [42,43]. The MND Australia data covers 1959–2013 and was originally compiled by a requested custom search of the AIHW General Record of Incidence of Mortality Books (GRIM). The raw data obtained from the search is presented in a supplemental file 1. The data used in the regression calculations for the time period between 1962 and 2013 and the statistical results are also presented in a supplementary file 1. The lagged forward 20 years regression could not include the years 1959–1961 because petrol air lead was unavailable for the war years in 1940 and 1941. The data includes, age standardised male death rate; female MND death rate; and percentage that MND was as a cause of all deaths in Australia. The percentage all cause MND death was originally calculated as the number of MND deaths per year divided by the total number of deaths per year. 

Taking into account the combination of age of exposure and the bone lead reservoir of exposure we assume a lag in the onset of MND response. The best fit of MND response lag was determined by performing linear regressions between accumulated petrol lead emissions between 1933 and 2002 with forward lags of 10 to 24 years for male and female MND death rates and for percent MND all cause death in Australia between 1962 and 2013. The best fit was 20 years for lag response because 20 years showed the largest *R^2^* value and smallest *p*-value. Twenty year lag of MND response was thus selected for further evaluation for accumulated petrol lead emissions.

## 3. Results

**Figure 2:**
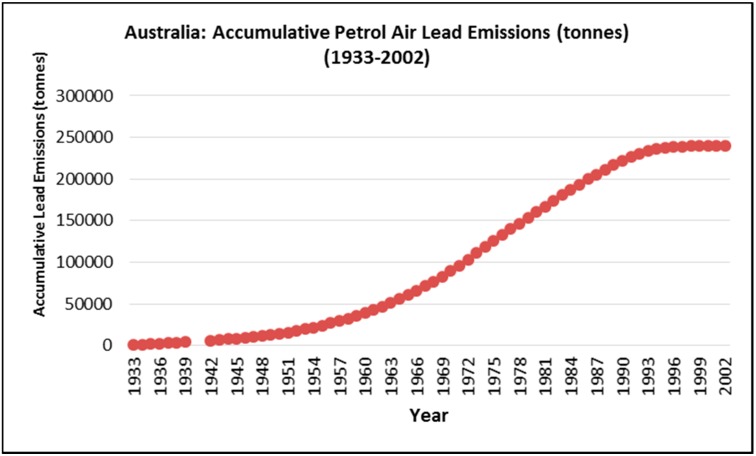
Accumulated lead emissions from leaded petrol combustion in Australia (1933–2002). (Data sourced from Kristensen, 2015 [40]).

The petrol lead emission trend calculated by Kristensen (2015) [40] was converted to petrol lead accumulation in Australia between 1933 and 2002 as shown in Figure 2. 

**Figure 3:**
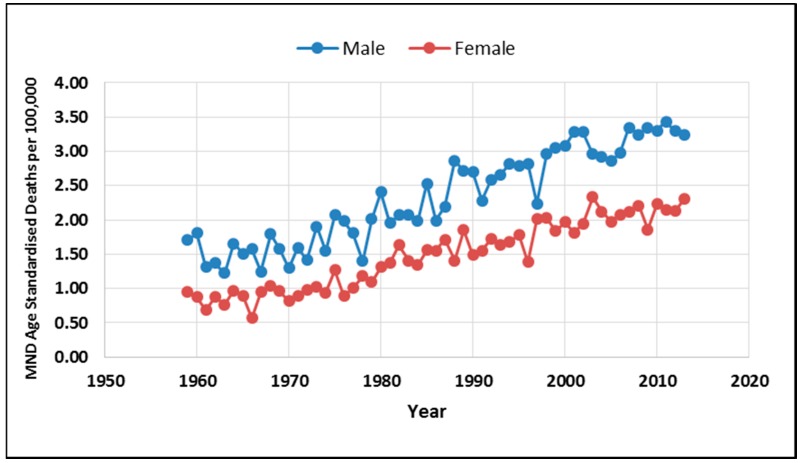
Age-standardized male and female MND death rate in Australia: 1959–2013.

As illustrate in Figure 3, the age-standardized male MND death rate in Australia increased 89.5% from 1.71/100,000 in 1959 to 3.24/100,000 in 2013 and the age-standardized female MND rate increased 143.2% from 0.95/100,000 in 1959 to 2.31/100,000 in 2013. This finding indicates a larger age-standardized death rate in males than females in Australia. 

**Figure 4:**
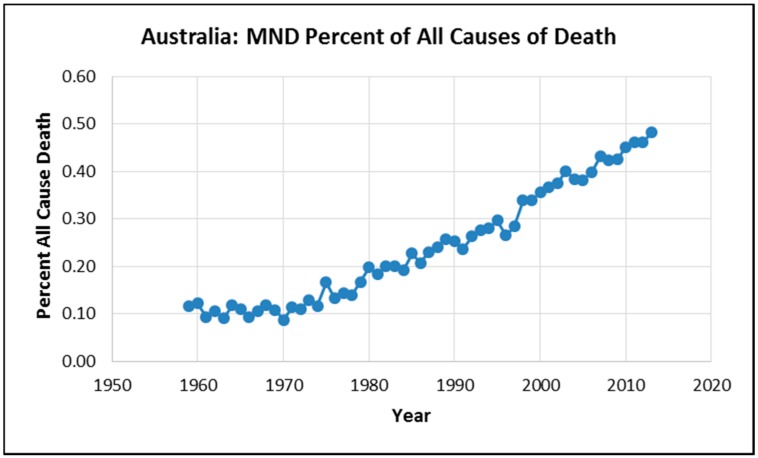
Percent all cause MND death percentage: 1959–2013.

The combined male and female percent all cause death attributed to MND increased 300% from 0.12% in 1959 to 0.48% in 2013 and this result is illustrated in Figure 4. 

**Figure 5:**
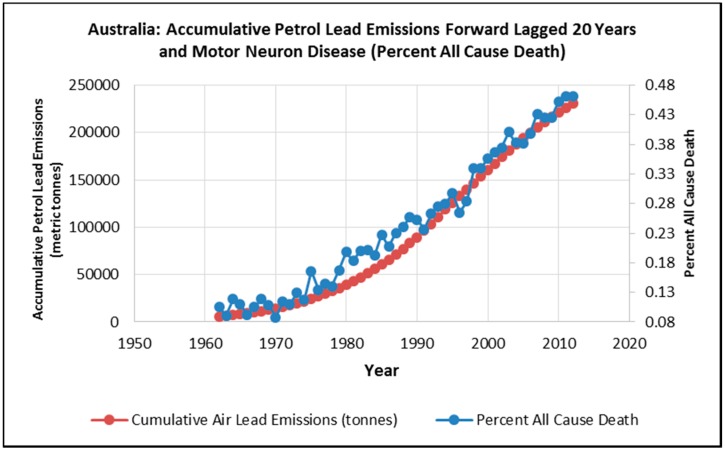
Twenty year forward lag of petrol lead emissions and motor neuron disease as a percent of all cause death for males and females in Australia.

Regression results between a 20 years lag of petrol lead emissions and age-standardized death rates indicate positive correlations for males (*R*^2^ = 0.96, *p* = 9.4 × 10^−23^). Likewise regression results between a 20 years lag of petrol lead emissions and age-standardized death rates indicate positive correlations for females (*R*^2^ = 0.96, *p* = 4.88 × 10^−23^). As illustrated in Figure 5 when male and female MND data are combined the regression results between a 20 years lag of petrol lead emissions and age standardized death rates indicate an extremely strong association for percent all cause death attributed to MND (*R*^2^ = 0.98, *p* = 2.6 × 10^−44^) . The data used in the regression analysis and the statistical results are presented in a supplementary file 2.

## 4. Discussion

### 4.1. Limitations and Additional Trend Rates

A major limitation of this study is that causality between accumulated petrol lead emission trends and MND trends in Australia cannot be proven with an ecological study design. This observational study design is an analytic time-trend ecologic study [44]. One potential limitation of ecologic studies is known as the ecologic fallacy: the failure of expected ecologic effect estimates to reflect biologic effects at the individual level. Thus, our results should be viewed with a degree of caution. 

There are other limitations of the study. Chio *et al.* (2000) [45] acknowledges that MND rates have been generally increasing internationally and reviews some of the explanations for the increase. Chio *et al.* (2000) [45] notes that based on numerous protocols of various European agencies of many nations the increase in the MND mortality could be attributed to increasing accuracy of the death certificates over time; however they conclude that this explanation is probably insufficient given that the increase in mortality has been consistent across many different countries with different health systems. The Australian database may not have the same limitations because, as described in Section 2, the AIHW establishes protocols and manages the database for the entire country. 

Chio *et al.* (2000) [45] also suggested that another explanation for the increase in MND mortality is that while the mean age of the population has increased (prolonged survival) this has caused diseases to compete with each other as a cause of the effect. For instance, the effect of a decline in mortality due to cardiovascular and cerebrovascular diseases may result in an increase in the mortality of less common diseases. This process has been named the “Gompertzian effect”. Furthermore, Chio *et al.* (2000) [45] suggest that there are other methodological problems with ALS data such as issues with case definition, case ascertainment, selection bias, heterogeneity of study design and difficulties in diagnosing sporadic/isolated cases of ALS early in the disease and in the presence of additional/atypical signs. Furthermore, in addition to lead, there may be other unknown environmental factors that are causally related to the development of MND that may be increasing in the environment over time. 

Another factor that had the potential to effect the international MND mortality trends was the international diagnostic criteria for ALS/MND diagnoses named the El Escorial diagnostic criteria which was implemented in the late 1990s for the purpose of standardising the steps of diagnosis, clarifying the complexity of various clinical features and ensuring diagnostic criteria. Agosta *et al.* (2015) [46] state that the literature before and after the publication of the El Escorial criteria did not provide an improvement regarding the accuracy of the diagnosis or diagnostic delay. Thus this suggests that the El Escorial criteria did not change the mortality death trend patterns after their introduction in the late 1990s.

It must be noted that there are other factors causing ALS, such as genetics in familial ALS [47], occupational lead exposure [48] and other environmental factors such as solvents [49], agricultural chemicals [50] pesticides [51] and cyanobacterial neurotoxins such as BMAA [52]. These factors could not be properly controlled in the calculations due to missing information and they may exert influence on the data validity and conclusions. It is conceivable that exposure to many of these environmental factors have also increased and therefore may contribute to the rise in MND mortality.

The introduction emphasized the increasing trend of MND mortality; however, there have also been some exceptions to the international trend of MND mortality rate increases. For examples, while Noonan *et al.* (2005) [9] observed an increase in MND mortality in the United States between 1969 and 1998, Mehal *et al*., (2013) [53] indicated that in the United States between 1999 and 2009 the annual rate for males decreased whereas the rate for females showed no change, however the ALS/MND associated death rate slightly increased among adults 20–49 years of age. While Noonan *et al.* (2005) [9] concluded that variations in motor neuron disease (MND) mortality by time, race/ethnicity, sex, and geography for the time period between 1969 and 1998 were consistent with the hypothesis that environmental exposures, combined with factors of genetic susceptibility, play a role in the development of MND, Noonan *et al.* (2005) [9] also noted that under diagnosis and death certificate reporting may be the explanation for the increase in mortality during that time period. In Olmstead County Minnesota (USA) Sorenson *et al.* (2002) [54] found that the incidence rate remained stable at 1.7 cases per 100,000 people per year between 1925 and 1998. This is, however, a small sample from Minnesota, rather than a national database. Contrasting results in Scotland were reported; Swingler *et al.* (1992) [55] observed that the standardised mortality ratio increased from 68 to 124 between 1968 and 1987 although Forbes *et al.* (2007) [56] noted that between 1989 and 1998 the MND mortality rate remained stable in Scotland with an annual standardised incidence of 2.40 per 100,000 (95% CI 2.22–2.58). In the United Kingdom, Alvaro *et al.* (2009) [57] found that between 1990 and 2005 no increase in MND incidence was apparent. The Australian trend may not be universal.

### 4.2. Possible Prediction of Future MND Death Trends in Australia 

We acknowledge that we cannot conclude that the association between the forward lag of petrol lead emissions are causally related. However if these variables were found to be causally related, then based on the plateau of the forward lag of the accumulated petrol curve at the end of the petrol lead accumulation curve (Figure 2) and the lagged relationship of MND percent all cause death (Figure 5), we predict that the mortality rate of MND in Australia may stabilise and then start to decline in the coming years.

### 4.3. Need for Further Study

Urban environments are the habitat for most of the world’s peoples and it is essential to fully understand the qualities which sustain life. Non-occupational lead exposure from the legacy of petrol lead emissions was a major contaminant introduced into urban environments worldwide and it provides a case example of the need for further study of processes that contaminate urban environments. The history of the use in petrol lead worldwide has been described as occurring in two phases. The first phase involved the rise in total lead emissions between the 1930s and the 1970s, and the second phase involved the phase down of lead in various countries between the 1970s and the present time [58]. After the 1970s different countries phased out petrol lead at different times [59]. In the United States, lead was gradually phased out of petrol in multiple steps starting with the 1975 requirement for unleaded petrol to protect the catalytic converter, the rapid phased down in 1986, and the total ban of lead additives in petrol for highway vehicles in 1996 [60,61,62]. In Australia lead was phased out of petrol in steps between the mid-1980s and 2002 [40].

We postulate that the petrol lead source and its legacy of aerosol emission and accumulation became a driver for increasing the incidence of MND. The lead emissions contaminate surface soil and are then resuspended into the atmosphere during dry periods with fine lead enriched soil dust migrating into homes [30,63,64]. Children are exposed through inhalation and ingestion via hand to mouth activity. Urban atmospheric lead concentrations are largest during dry seasons of the year [30,63]. The lead emissions into urban atmospheres are correlated with urban soil lead concentrations [30]. In addition lead contaminated soils are tracked into homes via shoes or pets [65,66]. Children’s blood lead levels have been associated spatially [67] with soil lead concentrations and temporally with atmospheric lead concentrations [68]. 

Multiple lines of evidence suggest that lead exposure plays a significant role in the aetiology of MND. These lines of evidence include the meta-analysis between MND and occupational lead exposure [11], previous bone lead studies [23,24,25,26] and studies that have found positive associations between MND and lead in human biological samples [13,14,16], questionnaires [12,20,21,22] and the results from this study. Lead is also known to cause oxidative stress [69,70]. Oxidative stress biomarkers have been observed in sporadic MND patients [71] and oxidative stress has been implicated in ALS pathogenesis [72]. While there is sufficient evidence that occupational lead exposure plays a role in the aetiology of MND the findings in this study also suggests that non-occupational exposure from lead additives in petrol plays a role in the aetiology of MND. Non-occupational exposure is derived from petrol lead emissions that accumulated over time in urban soils and house dusts and became incorporated into the bones of various cohorts of humans with the total bone lead dosage varying relative to the accumulated concentrations in the soils and dusts. Based upon the associations between petrol lead emissions and MND trends in this study, we hypothesize that MND develops approximately 20 years following the bioaccumulation of petrol derived lead in human bones.

### 4.4. Need for Further Study

Non-occupational lead exposure remains a pervasive and a continuing worldwide problem from air lead, soil lead and lead dust in homes, especially in urban environments. Primary prevention of exposure is paramount to solving the negative health outcomes associated with lead exposure. The Precautionary Principle as defined by the United Nations Educational, Scientific and Cultural Organisation (UNESCO) (2005) [73] established guidelines for actions required to diminish risks to humans or the environment. If lead exposure is related to MND as indicated for Australia then this disease is one more reason for taking concerted actions to reduce legacy lead contamination.

## 5. Conclusions 

The emissions from leaded petrol have left a legacy in the environment. The legacy shows up as many forms of chronic diseases in the population. Diseases in the population can be both from early childhood exposure and later exposure during adulthood resulting in the accumulated storage of lead in bones and its subsequent release later in life. Regression results indicate positive correlations between a 20 year lag of petrol lead emissions and MND trends in Australia. The results indicate that non-occupational exposures to the population from past Australian petrol lead emissions and its environmental accumulation may have contributed to the development of sporadic MND. We predict that when evaluated in other countries similar findings will also be found. Further examination of a lag between exposure to petrol lead accumulation in urban soils and dusts and MND trends is warranted. If these studies confirm that exposure to lead from past emissions of lead in petrol is causally related to the development of MND, then lead contaminated urban soils and house dusts may require extensive remediation or isolation to prevent further development of sporadic MND in future generations. 

## Supplementary Files

Supplementary File 1

Supplementary File 2
